# Supplementary figures and images for: Changes in Multiple microRNA Levels with Antidepressant Treatment Are Associated with Remission and Interact with Key Pathways: A Comprehensive microRNA Analysis
Source: Int J Mol Sci. 2023 Jul 30;24(15):12199. doi: 10.3390/ijms241512199 (PMC10418406; doi:10.3390/ijms241512199)

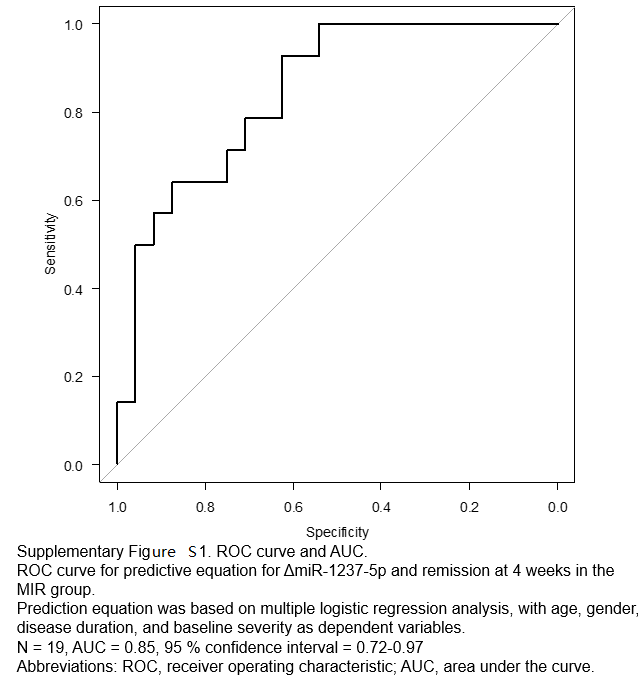

Supplement: Supplementary file 1 [file ijms-24-12199-s001.zip › Supplementary Figure S1.PNG]

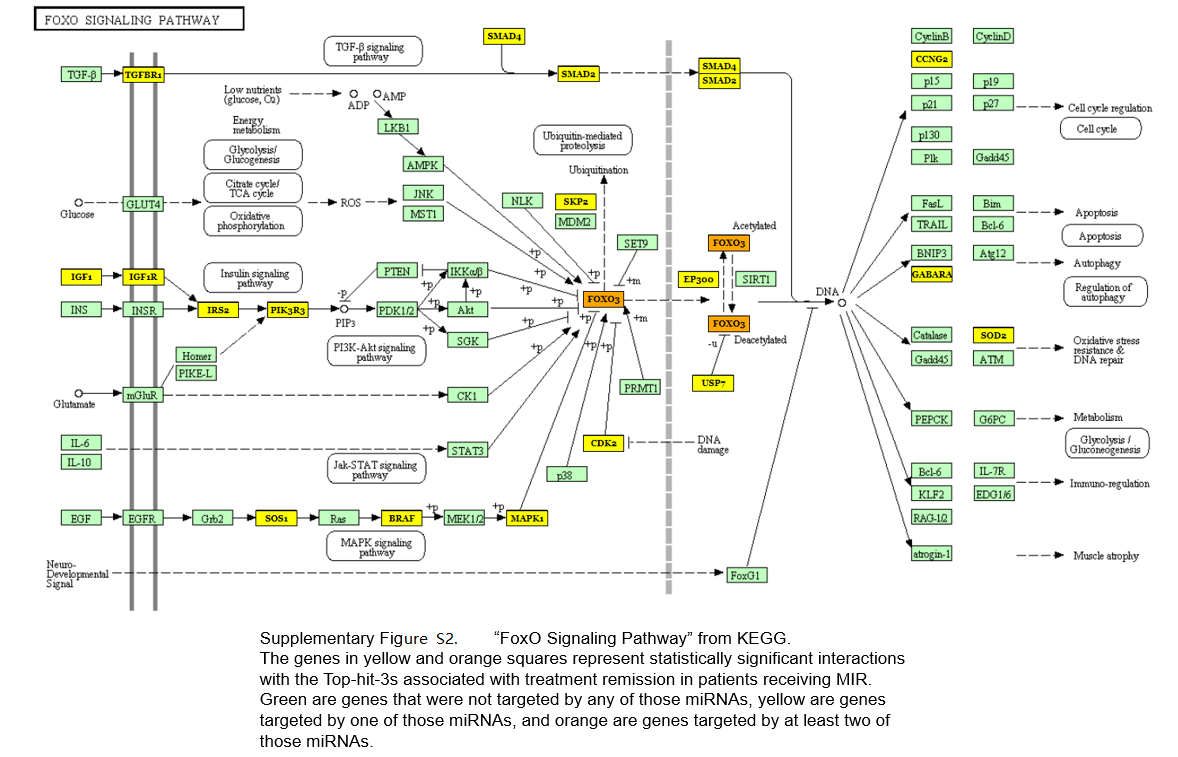

Supplement: Supplementary file 1 [file ijms-24-12199-s001.zip › Supplementary Figure S2.PNG]

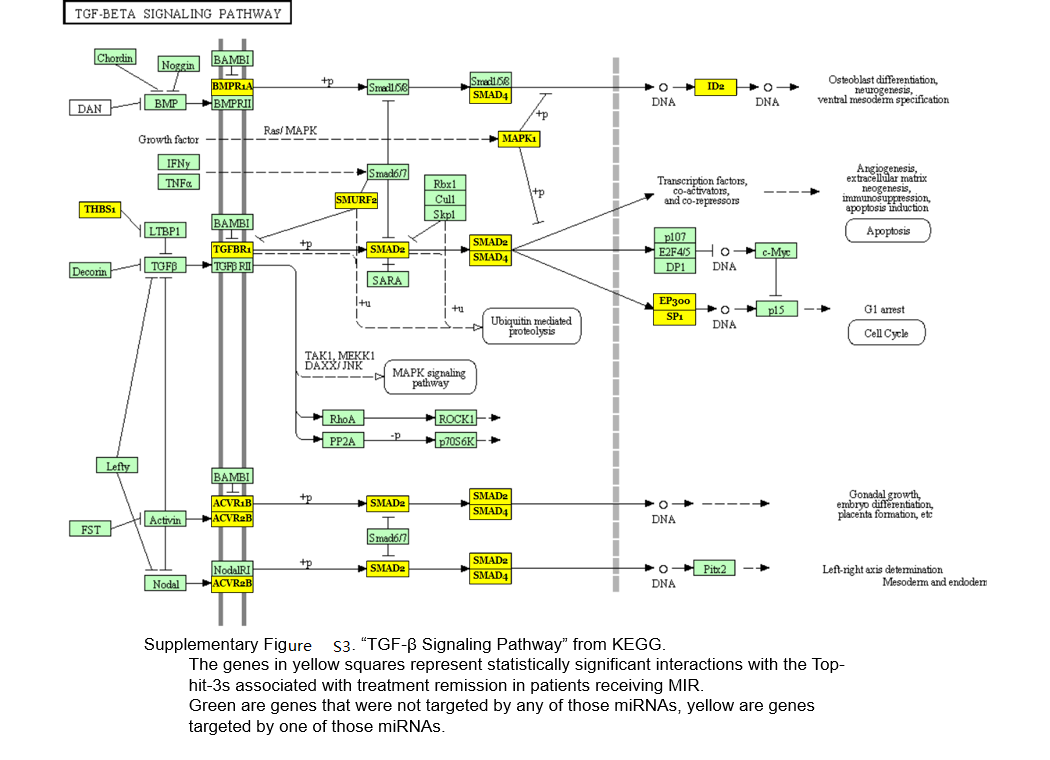

Supplement: Supplementary file 1 [file ijms-24-12199-s001.zip › Supplementary Figure S3.PNG]

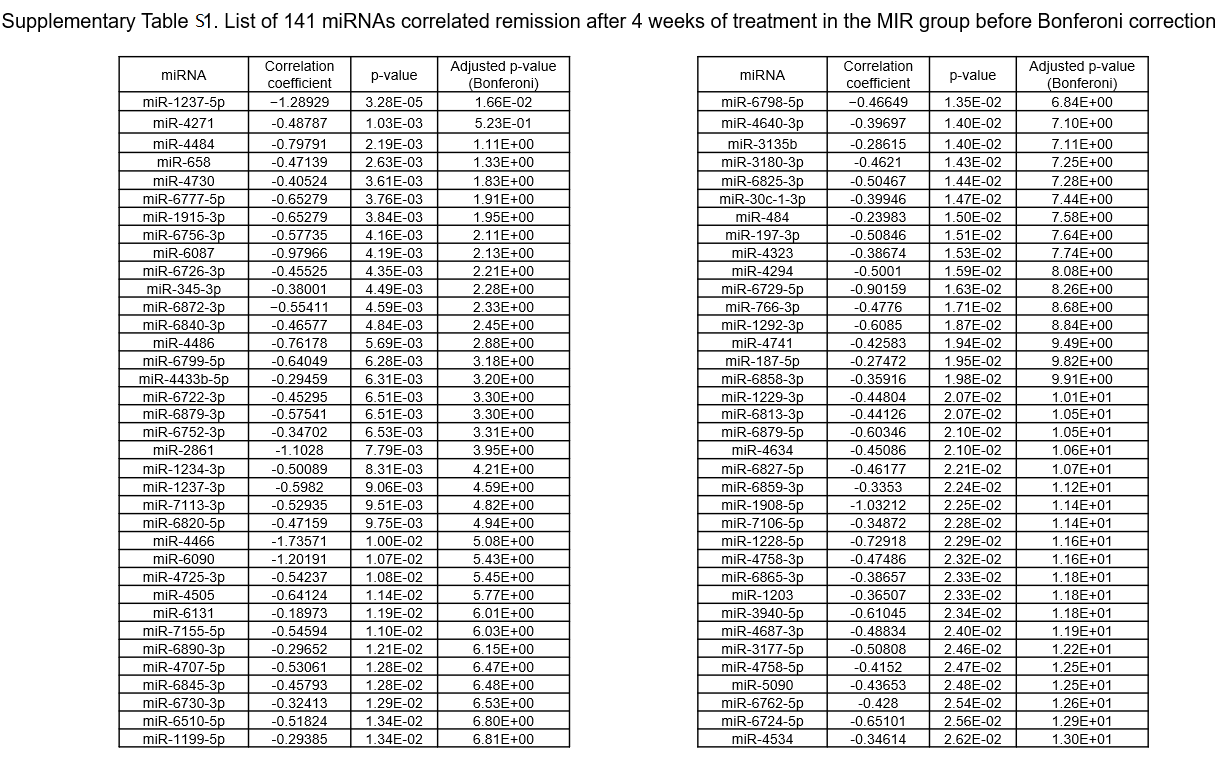

Supplement: Supplementary file 1 [file ijms-24-12199-s001.zip › Supplementary Table S1-1.PNG]

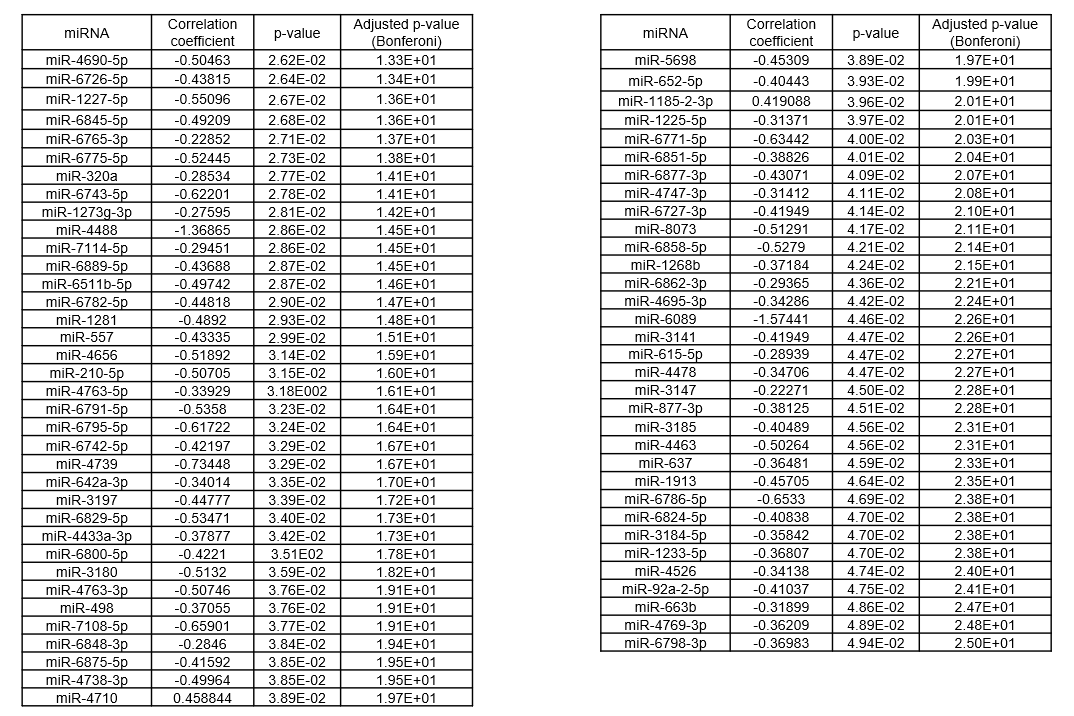

Supplement: Supplementary file 1 [file ijms-24-12199-s001.zip › Supplementary Table S1-2.PNG]
